# From "Laughing Gas" to "Galaxy Gas": An Updated Review on the Neurological, Pathologic, and Psychiatric Sequelae of Nitrous Oxide Abuse

**DOI:** 10.7759/cureus.93608

**Published:** 2025-09-30

**Authors:** Aditya Lal Vallath, Ratan P Yadav, Michelle Bass, Rashmitha Pippari, David Gnugnoli

**Affiliations:** 1 Emergency Medicine, Conemaugh Memorial Medical Center, Johnstown, USA; 2 Internal Medicine, Conemaugh Memorial Medical Center, Johnstown, USA; 3 School of Medicine, Universidad El Bosque, Bogota, COL

**Keywords:** emergency medicine, n2o abuse, public health, substance abuse, toxicology

## Abstract

Nitrous oxide (N₂O), a dissociative anesthetic and analgesic, has seen a concerning rise in recreational abuse, particularly among adolescents and young adults. This resurgence is fueled by its easy accessibility in large-volume, flavored canisters marketed as cooking aids, which exploit legal loopholes, as well as its promotion through popular culture and social media platforms like TikTok and YouTube. This literature review synthesizes current knowledge on N₂O abuse by systematically searching PubMed, Embase, Scopus, Web of Science, and PsycINFO for articles published from January 1, 2015, to February 3, 2025. The review focuses on the pathophysiology, symptoms, and long-term effects of N₂O abuse in both adult and pediatric populations, including its physical, psychiatric, and addiction-related consequences.

The review of 13 studies, which included retrospective cohorts, case series, and cross-sectional surveys, highlights the severe neuropsychiatric and physical consequences of N₂O abuse. Epidemiological data consistently show that use is highest among young adults aged 16-30 years, with a notable overrepresentation of males and a disproportionate impact on socioeconomically disadvantaged populations. The most prominent physical effect is subacute combined degeneration of the spinal cord (SCD), which is a spinal cord disorder caused by demyelination of its lateral and posterior columns. It typically leads to neurological symptoms with common signs including numbness, tingling, muscle weakness, coordination problems, and difficulty walking. Biochemically, N₂O abuse leads to a functional vitamin B₁₂ deficiency, marked by elevated homocysteine and methylmalonic acid levels. Psychiatrically, N₂O use is strongly associated with a range of symptoms, including anxiety, depression, and psychosis. The review also notes patterns of compulsive use and polydrug abuse, which suggests a high potential for dependence.

This review underscores the urgent need for heightened clinical suspicion of N_2_O abuse among healthcare professionals. Public health recommendations include enhanced regulation of N₂O sales, clearer product labeling, and targeted social media campaigns such as collaborating with popular content creators and public service announcements to combat misinformation about its safety. The “Galaxy Gas” trend, where individuals, particularly in schools and public spaces, purchase and misuse large N₂O canisters promoted on social media, warrants specific attention due to its broad reach and ease of access. Future research should prioritize on understanding the long-term neuropsychiatric impacts of N₂O and developing effective treatment strategies. Notably, recent progress has been made with health agencies enforcing age restrictions on the sale of high-volume N₂O canisters, including bans for those under 21.

## Introduction and background

Nitrous oxide (N₂O), a colorless gas with a slightly sweet odor, has a long and storied history in medicine, dating back to its discovery in the late eighteenth century. Initially celebrated for its euphoric and analgesic properties, it rapidly became a staple in surgical and dental procedures. Decades of research have elucidated its pharmacodynamic effects, primarily mediated through NMDA (N-methyl-D-aspartic acid) receptor antagonism and GABAergic (gamma-aminobutyric acid) potentiation [1]. However, despite this extensive understanding of its therapeutic applications and known risks, a concerning resurgence in N₂O abuse has emerged, fueled by the widespread availability of large-volume, flavored canisters marketed under brands such as "Galaxy Gas." These products, often sold under the guise of culinary or recreational use, have facilitated easy access to high concentrations of N₂O, particularly among younger populations [2-4].

This review aims to comprehensively explore the contemporary landscape of N₂O abuse, delving into its abuse potential, intricate pathophysiological mechanisms, and the spectrum of short- and long-term sequelae. Notably, it will place a significant emphasis on the unique vulnerabilities and consequences of N₂O abuse within the child and adolescent demographic, a population particularly susceptible to the allure of readily available psychoactive substances. By synthesizing current literature, this review seeks to provide a critical analysis of the evolving challenges posed by N₂O abuse, highlighting the urgent need for targeted interventions and public health initiatives to mitigate its detrimental impact.

## Review

This literature review aimed to comprehensively synthesize current knowledge regarding N₂O abuse, focusing on its physical effects (both short and long term), psychiatric effects, and addiction potential. To achieve this, a systematic search strategy was employed across multiple electronic databases. This review also follows "Preferred Reporting Items for Systematic Reviews and Meta-Analyses" guidelines.

Search strategy

A thorough search was conducted across PubMed, Embase, Scopus, Web of Science, and PsycINFO. The search utilized a combination of keywords and Medical Subject Headings (MeSH) terms, including "Nitrous Oxide," "N₂O," "Nitrous Oxide Abuse," "Recreational Nitrous Oxide," "Nitrous Oxide Toxicity," "Nitrous Oxide Neuropathy," "Vitamin B12 Deficiency," "Subacute Combined Degeneration," "NMDA Antagonist," "GABAergic," "Psychiatric Effects," "Addiction," "Dependence," "Cognitive Impairment," "Adolescent," "Child," "Short term abuse," "Long term abuse," and "Galaxy gas." Search strings were customized for each database, combining these terms with Boolean operators (AND, OR, NOT) and truncation symbols (*). The search period covered January 1, 2015, to February 3, 2025. While the primary focus was on studies published in English, relevant studies in other languages with English abstracts were also considered. Additionally, gray literature was sought through Google Scholar, news publications, and relevant organizational websites such as the National Institute on Drug Abuse and the World Health Organization to identify reports, guidelines, and conference proceedings.

Inclusion and exclusion criteria

Inclusion criteria for the review included studies that examined the physical and psychiatric effects of recreational N₂O abuse. Eligible studies addressed both short-term and long-term physical health consequences, as well as a range of psychiatric outcomes, including mood disorders, psychosis, and cognitive impairments. Particular attention was given to studies exploring the addiction potential and patterns of dependence associated with N₂O use, especially among vulnerable populations such as adolescents and children. A wide range of study designs was considered, including case reports, observational studies, clinical trials, and systematic or narrative reviews, to capture both clinical insights and broader epidemiological trends.

Conversely, studies were excluded if they focused exclusively on the therapeutic use of N₂O in medical or dental contexts, such as anesthesia or analgesia, without addressing recreational misuse. Research that dealt solely with industrial or environmental applications of N₂O was also excluded. Additionally, studies that did not specifically investigate the harms or behavioral effects of recreational N₂O use or that lacked relevance to the review’s core objectives were omitted. Lastly, papers that were not accessible via standard academic databases or institutional access (e.g., paywalled without availability through interlibrary systems) were not considered for inclusion.

Study selection and data extraction

The study selection process involved two independent reviewers who screened the titles and abstracts of retrieved articles for relevance. Full-text articles of potentially eligible studies were then retrieved and assessed against the predefined inclusion and exclusion criteria. Any discrepancies between reviewers were resolved through discussion and consensus.

For data extraction, a standardized form was used to collect information from the included studies. This data included study design, demographic characteristics (age, sex, and population), N₂O exposure details (frequency, duration, and route of administration), physical effects (neurological, cardiovascular, respiratory, and hematological), psychiatric effects (mood, cognition, and psychosis), addiction potential and dependence, diagnostic criteria used, and treatment and management strategies.

Quality assessment and data synthesis

The quality of included studies was assessed using appropriate critical appraisal tools, such as the Newcastle-Ottawa Scale for observational studies and specific tools for case reports such as Joanna Briggs Institute (JBI) Critical Appraisal Tools. The risk of bias for each study was assessed and noted within the review.

Finally, a narrative synthesis of the findings was conducted, organizing the extracted data into key themes: short-term physical effects of N₂O abuse, long-term physical effects of N₂O abuse, psychiatric effects of N₂O abuse, addiction potential and dependence, and effects on child and adolescent populations. Where available, quantitative data were summarized and presented in tables or figures. Heterogeneity across study designs and outcomes was addressed through a descriptive analysis.

Results

This literature review summarizes the findings of 13 studies, incorporating a range of methodological designs, including retrospective cohort studies, case series, cross-sectional surveys, and individual case reports (Table 1).

**Table 1:** Summary of 13 included studies on nitrous oxide (N₂O) abuse, including study design, sample characteristics, key findings, and limitations N₂O: Nitrous oxide; SCD/SACD: Subacute combined degeneration; ED: Emergency department; EMG: Electromyography; MMA: Methylmalonic acid; KAP: Knowledge, attitudes, and practices; AIDP: Acute inflammatory demyelinating polyneuropathy; SCL-90: Symptom Checklist-90.

Author (Year, Country) [Ref]	Study Design/Sample	Key Findings	Limitations
Dawudi et al. (2024, France) [1]	Retrospective multicenter cohort (181 N₂O-related neurological cases, adults, 2018–2021)	25% myelopathy, 37% neuropathy, 38% mixed disease; peak incidence 2021; findings suggest a strong socioeconomic gradient in N₂O-related neurological morbidity (20–25 years).	Hospital-based cohort; multicenter but limited to Greater Paris; possible underestimation of community cases.
Bethmont et al. (2019, Australia) [2]	Retrospective ED surveillance (118 cases, 60 hospitals)	Between 2016 and 2018, presentations to NSW emergency departments for illicit nitrous oxide use rose sharply, with most patients (83%) being young adults aged 16-30. Half of the cases involved polydrug use. The presentations included a variety of health issues, with neurological (12%), self-harm (14%), and mental health (24%) issues being prominent.	Letter to editor, lacked robust methods, ED-based only; lacks outpatient/community data; only reports patients seeking care, which may underrepresent the true number of cases; limited follow-up.
Dai et al. (2024, China) [3]	Single case report with narrative literature synthesis (one patient, five-year follow-up; 84 articles reviewed)	Case: MRI “inverted V” sign → SCD; full recovery after B12-based therapy. Literature shows rising global N₂O abuse.	Single case; narrative review design; limited generalizability.
Winstock and Ferris (2020, UK/Global) [4]	Cross-sectional survey (Global Drug Survey 2014–2016; 16,124 users)	3.3% reported persistent paresthesia; dose–response relationship; neuropathy risk rises with heavy use.	Self-reported survey; recall bias; potential for underestimation due to sampling bias; no diagnostic confirmation.
Snyder and Howard (2015, USA) [5]	Cross-sectional survey (723 incarcerated youth, aged 13–17)	N₂O and other volatile solvent users showed higher rates of psychoticism, obsessive-compulsive traits, suicidal ideation, and neurological history. Also noted, psychiatric comorbidities may be exacerbated in high-risk youth populations.	Restricted to incarcerated adolescents; findings may not generalize to community youth; older dataset.
Wu et al. (2022, China) [6]	Observational hospital case series during COVID-19 lockdown (6 young adults; May–June 2020)	SCD-type neurological deficits; numbness/ataxia, MRI “inverted V” (4/6), EMG neuropathy (5/6), ↓ B12, ↑ homocysteine; severe anxiety/depression on SCL-90; improved after B12.	Single center, small sample; no concurrent citywide control data; observational design limits causal inference.
Riccò et al. (2023, Italy) [7]	Cross-sectional physician survey (KAP study)	Survey revealed limited physician awareness of N₂O-related neuropsychiatric risks.	Survey of physicians, not patients; clinical applicability limited. Limits insight into actual clinical presentations and outcomes.
Einsiedler et al. (2021, France) [8]	Case series (five patients, aged 19–29)	Four cases of SACD, one AIDP; ↑ homocysteine and MMA despite normal B12; MRI/EMG confirmed findings; B12 therapy led to partial–full recovery.	Small case series; limited generalizability; descriptive only.
Kulkantrakorn et al. (2024, Thailand) [9]	Retrospective case series (seven patients, aged 19–32)	Cases of myelopathy and polyneuropathy; MRI and EMG are helpful for diagnosis.	Small case series; single center; limited generalizability; descriptive only.
Hawkins et al. (2024, UK) [10]	Cross-sectional survey (6,672 adolescents, 244 schools; 5.1% reported N₂O use)	N₂O and volatile use linked to depression, anxiety, and hallucinations.	Self-reported mental health data; causality cannot be established.
Kaar et al. (2016, UK/US/Germany) [11]	International online survey (4,883 respondents)	Lifetime use prevalence: UK 38.6%, US 29.4%, Germany 21%.	Self-reported survey; risk of recall/selection bias; no clinical confirmation.
Jiang et al. (2021, China) [12]	Retrospective hospital study (63 patients, aged 15–33)	100% sensory neuropathy, 97% gait disturbance; 63% depression, 60% anxiety; ↑ homocysteine in 87%, low/normal B12; MRI changes are more likely with early presentation.	Single-hospital cohort; no control group; limited generalizability.
Cao et al. (2021, China) [13]	Case reports (two patients, females, aged 19–20)	Cerebellar atrophy and low copper were observed alongside B12 deficiency.	Two cases only; small sample; no long-term outcomes.

**Figure 1:**
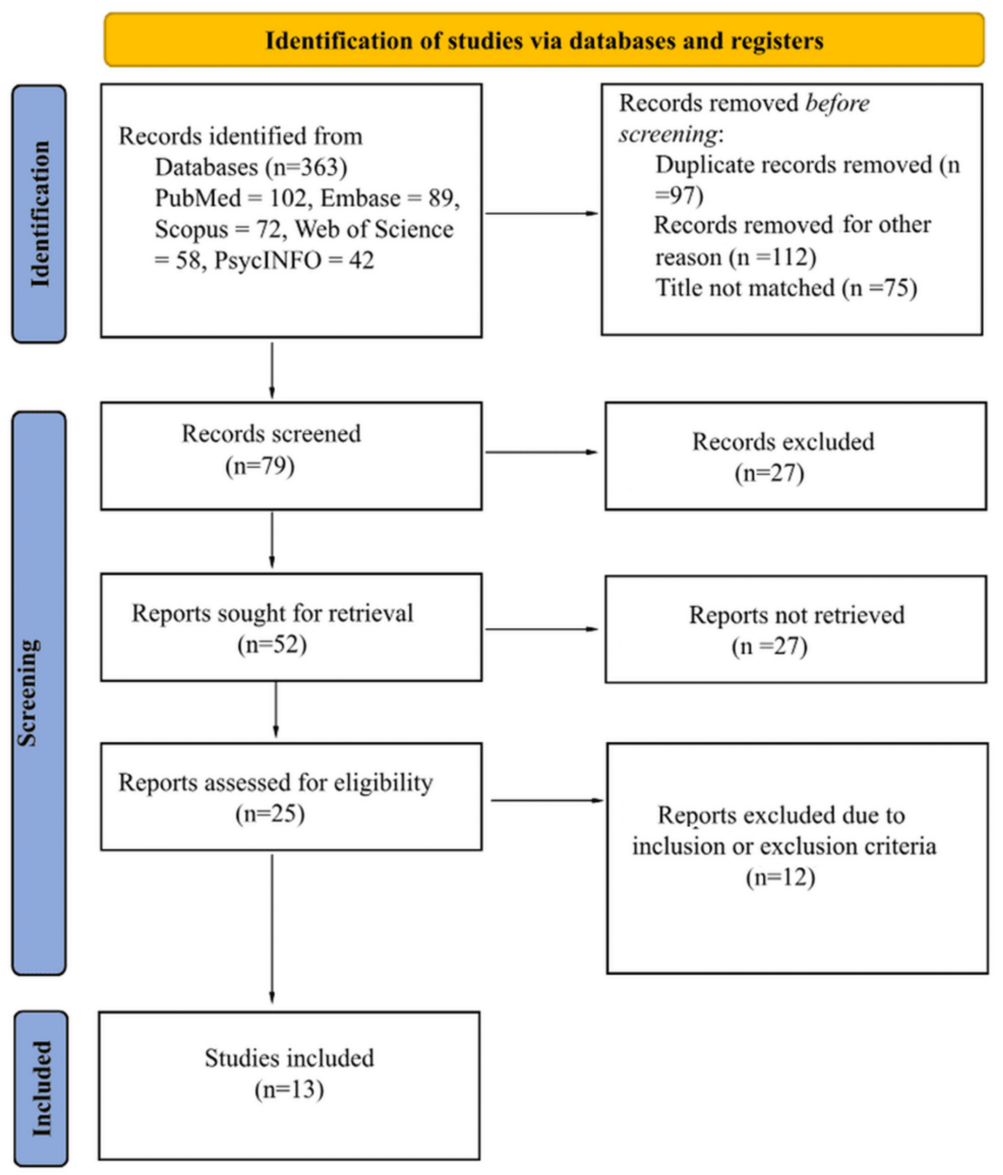
PRISMA flow diagram The flow diagram depicts the flow of information through the different phases of a systematic review.

Collectively, these studies shed light on the epidemiology, symptomatology, diagnostic methods, and management techniques associated with N₂O abuse (Figure 1). Given the diverse nature of these studies varying in design (e.g., case reports, cohort studies, and cross-sectional surveys), populations (e.g., adults, adolescents, clinical vs. nonclinical), and reported outcomes (e.g., neurological, psychiatric, and biochemical findings), a meta-analysis was deemed inappropriate. Instead, this review provides a descriptive analysis of the included studies, highlighting variations in study designs, population characteristics, clinical outcomes, and diagnostic and treatment methods. This narrative synthesis focuses on identifying recurring patterns and unique findings across the studies, acknowledging the diversity in approaches and reporting standards.

Epidemiology and User Profile

The studies consistently show that N₂O consumption is more common among adolescents and young adults, typically aged 16-30 years, with a particular concentration in those aged 20-25 years [1-5]. Most samples were slightly overrepresented by males, and users often came from socioeconomically disadvantaged backgrounds or were incarcerated [6-13]. Kaar et al. [11] reported the highest lifetime N₂O use internationally in the United Kingdom (38.6%), the United States (29.4%), and Germany (21.0%). In the United Kingdom, 5.1% of 13-14-year-old schoolchildren reported lifetime N₂O use [10].

Dawudi et al. [1] examined 91,000 hospital records and identified 181 N₂O-induced neurological illnesses (NI-NDs). Of these, 38% involved both central and peripheral neuropathy, while 37% were peripheral neuropathy, and 25% were myelopathy. In individuals aged 20-25 years, the incidence of N₂O-induced myelopathy was 6.15 per 10,000 in 2021, and peripheral neuropathy was 7.48 per 10,000. These figures are significantly higher than the incidence of non-N₂O myelitis (0.35/10,000) and Guillain-Barré syndrome (2.47/10,000). Notably, the incidence of NI-ND was two to three times more frequent in socioeconomically deprived districts. Bethmont et al. [2] observed a linear increase in emergency department (ED) visits between 2016 and 2018 in New South Wales, Australia, with 118 cases reported across 60 EDs. Of these cases, 46% involved polydrug use, and 83% of patients were aged 16-30 years. Cross-national survey data [11] indicated that 38.6% of UK respondents and 29.4% of US respondents reported using N₂O at least once. Currently, there is an ongoing debate about how to classify N₂O as a drug of abuse. While some researchers argue it lacks the classic withdrawal symptoms and reinforcing properties of other addictive drugs, others believe it should be considered an inhalant use disorder. This is due to the harmful behavioral patterns and serious health consequences, such as neurological damage, that are seen in frequent users.

Psychiatric and Psychological Effects

Short-term N₂O abuse consistently correlated with psychiatric symptoms. Jiang et al. [12] found that among inpatients, 60.3% experienced anxiety, and 63.5% developed depression. Snyder and Howard [5] reported that incarcerated youth (N = 723) who abused N₂O and volatile substances scored significantly higher on the Brief Symptom Inventory, showing elevated psychoticism and obsessive-compulsive traits. N₂O use was also significantly associated with probable depression (OR = 1.77), anxiety (OR = 1.59), auditory hallucinations (OR = 1.50), and conduct disorder (OR = 2.51), even after accounting for sociodemographic and behavioral confounding factors [10].

Physical and Neurological Effects

The most prominent and frequently reported physical effects of long-term N₂O abuse were neurological complications. Several case series and reports [3,8,9] described typical manifestations of subacute combined degeneration (SCD) of the spinal cord, including numbness, gait instability, proprioceptive losses, Lhermitte's sign, and distal weakness. A common radiological finding was the inverted V appearance on MRI due to dorsal column hyperintensities, particularly at the thoracic spinal levels [3,4,13]. Electrophysiological investigations often revealed sensorimotor polyneuropathies, both demyelinating and axonal [11]. Severe complications, such as respiratory arrest and altered consciousness, were also reported, especially among polydrug users requiring ED admission [2]. These findings highlight the potential for neurotoxicity with chronic or high-frequency N₂O exposure.

Addiction and Patterns of Use

While formal diagnostic criteria for substance use disorder were not consistently applied across studies, some evidence suggested patterns associated with psychological dependence and habitual use [4]. Wu et al. [6] as well as Snyder and Howard [5] noted that incarcerated youth reported using N₂O for over 100 days. Jiang et al. [12] observed an average frequency of 3.33 sessions per week, and Einsiedler et al. [8] documented individuals inhaling up to 50 cartridges in a single day. Although addiction was not always the primary focus, these recurring patterns, often accompanied by polydrug use, suggest the potential for dependence and compulsive behavior [9]. It has largely been fueled by social media and the widespread availability of products like “Galaxy Gas.” While seemingly innocuous, these flavored whipped cream chargers are being misused for their intoxicating effects, leading to severe health consequences, particularly among adolescents and young adults.

The ease of access is a major concern, as these products are inexpensive and readily available online through major retailers like Walmart and Amazon, often without strict age verification. The online sphere, especially platforms like TikTok and YouTube, plays a significant role in promoting this abuse. Videos depicting young people inhaling the gas, often as part of challenges or social dares, contribute to the normalization of N₂O misuse. This online exposure, combined with the false perception that N₂O is harmless due to its medical and culinary uses, creates a dangerous environment where teens and young adults are led to believe they are engaging in a "safe" recreational activity.

Diagnostic Tools and Biomarkers

In clinical studies, diagnostic methods largely comprised a combination of imaging, biochemical, and neurophysiological tests. MRI findings, especially posterior column hyperintensities, were strongly linked to clinical SCD [6,9]. Elevated levels of biochemical markers like homocysteine and methylmalonic acid (MMA) were common, sometimes even when serum vitamin B12 levels were normal or high [8,12]. This functional vitamin B12 deficiency proved crucial for diagnosis in cases where conventional measures might be misleading [1]. Other biomarkers, including low copper levels and cerebellar atrophy, were identified in some cases [13], suggesting a broader range of N₂O toxicity than commonly believed. In contrast, survey studies [4, 7] relied on subjective symptom data without laboratory or imaging confirmation, which can pose diagnostic challenges and introduce recall bias.

The reviewed literature underscores the pressing public health issue of recreational nitrous oxide (N₂O) use by adolescents and young adults. The findings consistently indicate that N₂O abuse leads to severe neuropsychiatric and physical consequences. Frequently observed complications include SCD, sensorimotor polyneuropathy, and psychiatric conditions such as depression, anxiety, and hallucinations [6,14]. A commonly encountered biochemical abnormality is functional vitamin B12 deficiency, which can be masked by normal or even high serum concentrations, highlighting the importance of secondary indicators like homocysteine and MMA in diagnostic panels [1,15].

Diverse diagnostic approaches were employed, with MRI, electromyography (EMG), and biochemical tests providing strong evidence in severe or chronic cases [2,13]. The characteristic inverted V sign in the dorsal spinal columns, along with elevated MMA and homocysteine, remains a key indicator of N₂O neurotoxicity. However, several case series reported considerable diagnostic delays, often attributed to low clinician awareness and underreporting of inhalant use. Positive treatment outcomes were observed with high-dose vitamin B12 supplementation in early stages or when combined with adjuvant treatments like folic acid, physiotherapy, and IVIg [1]. Recovery times varied from a few weeks to over a year, emphasizing the critical role of early screening and treatment [16,17]. Based on the evidence, several clinical and public health recommendations emerge. Emergency or primary care physicians should maintain a high index of suspicion for N₂O toxicity in young patients presenting with unexplained neurological or psychiatric complaints, particularly if polydrug use is involved [2,8]. A comprehensive history should include questions about inhalant use. In suspected cases, MMA and homocysteine levels should be ordered, even if B12 levels appear normal [6,16,18]. All medical professionals, especially those in emergency medicine, psychiatry, and neurology, require enhanced training to recognize and differentiate N₂O-related presentations from other neurological and psychiatric illnesses. Finally, long-term studies are needed to improve our understanding of chronic impacts, dependency risks, and optimal therapeutic regimens.

Geographically, the burden of N₂O abuse is most pronounced in high-income nations like the United Kingdom, France, Australia, and the United States, where recreational use is prevalent due to N₂O's ready availability. Socioeconomically disadvantaged groups and incarcerated individuals are particularly susceptible to abuse, pointing to a sociostructural dimension to these patterns. Furthermore, N₂O use is often promoted within young subcultures, and polydrug use frequently exacerbates clinical presentations [16,17]. Recently, online trends such as the abuse of “ Galaxy Gas,” marketed as flavored whipped cream chargers, have become a catchall term for N₂O products misused for their euphoric effects, primarily by adolescents and young adults. Increased regulation of N₂O sales and clearer labeling of potential harms could serve as effective harm-reduction strategies [19]. Public health messages, particularly via social media, are crucial to counteract misinformation regarding N₂O's perceived harmlessness [20]. Recently, an FDA report highlighted the increasing rise of abuse among adolescents and young adults after the release of Galaxy Gas in 2021 [21].

The "Galaxy Gas" trend has gained significant traction through social media platforms like TikTok, YouTube, and X (formerly Twitter). Hundreds of videos depict young people inhaling the gas, often as part of challenges or social dares. Influencers, video game streamers, and musicians have inadvertently or directly contributed to the normalization of N₂O abuse by featuring it in their content. This online exposure, coupled with bright, appealing packaging and enticing flavors (e.g., vanilla cupcake and blueberry mango), is explicitly designed to attract younger demographics, even if the products are ostensibly marketed for culinary use [22].

The accessibility of "Galaxy Gas" is a major contributing factor to its widespread abuse. Despite being sold as whipped cream chargers, these products are inexpensive and readily available online through major retailers like Walmart, Amazon, and eBay, often circumventing age restrictions. This ease of access, combined with the misconception that N₂O is harmless due to its medical and culinary applications, fuels the perception of it as a "safe" party drug. Lawsuits have been filed against SBK International, the company behind Galaxy Gas, alleging it marketed nitrous oxide canisters to young people while downplaying serious health risks such as nerve damage and even death. In response to growing concerns, Alabama passed SB 78, a law criminalizing the misuse and sale of nitrous oxide outside of approved settings. A 2025 class-action lawsuit claims the company used deceptive packaging and social media to target youth, contributing to widespread misuse and addiction [23-25].

Limitations

This review has several limitations. First, this review faces several methodological challenges; the diversity of study designs, sample sizes, and populations makes direct comparison of findings challenging. Many cross-sectional surveys rely on self-reported data, which can lead to underestimation of actual prevalence rates due to recall or social desirability bias. While clinically valuable, case reports and small case series may not represent the full spectrum of N₂O-related harm.

Additionally, a majority of the studies were conducted in Western countries, introducing geographic bias and limiting the applicability of findings to low-income or underrepresented regions. Furthermore, the scarcity of long-term data on the neuropsychiatric effects of N₂O use hampers our understanding of its chronic consequences and restricts the ability to draw conclusions about its sustained impact over time.

## Conclusions

A growing body of recent evidence highlights N₂O abuse as an emerging epidemiological challenge, carrying significant neurological and psychiatric consequences, particularly for adolescents and young adults. Despite N₂O's reputation as a relatively harmless recreational drug, frequent or heavy use can result in SCD, sensorimotor neuropathy, mood disorders, and cognitive dysfunctions, often stemming from a functional vitamin B12 deficiency. While early diagnosis, appropriate biochemical testing, and vitamin supplementation can lead to substantial recovery, diagnostic delays are common due to underreporting and low clinical awareness. Addressing this increasingly unrecognized and growing substance misuse phenomenon requires concerted efforts in clinical training, public health policy planning, and harm mitigation responses.
